# Colorectal Cancer in Inflammatory Bowel Disease: How to Minimize the Risk

**DOI:** 10.3390/cancers18203272

**Published:** 2026-10-10

**Authors:** Boris Nassera, Lucia Centanni, Ferdinando D’Amico, Alessandra Zilli, Tommaso Lorenzo Parigi, Federica Furfaro, Virginia Solitano, Mariangela Allocca, Silvio Danese

**Affiliations:** 1 Department of Gastroenterology and Endoscopy, IRCCS San Raffaele Hospital, 20132 Milan, Italy; nassera.boris@hsr.it (B.N.); damico.ferdinando@hsr.it (F.D.);; 2 Department of Gastroenterology and Endoscopy, Faculty of Medicine and Surgery, Vita-Salute San Raffaele University, 20132 Milan, Italy

**Keywords:** inflammatory bowel disease, colorectal cancer, treat-to-target, dysplasia, surveillance, prevention

## Abstract

Patients with inflammatory bowel disease (IBD) have a higher risk of developing colorectal cancer (CRC) compared with the general population. This increased risk is mainly related to chronic colonic inflammation and is influenced by factors such as disease duration, disease extent, family history of CRC, and the presence of primary sclerosing cholangitis (PSC). Although the incidence of CRC in IBD has decreased over recent decades, it remains an important cause of disease-related morbidity and mortality. CRC prevention in IBD extends beyond colonoscopic surveillance and includes high-quality endoscopic surveillance, early detection and appropriate treatment of dysplasia, and long-term control of intestinal inflammation through a personalized approach. This review summarizes current evidence on IBD-associated colorectal carcinogenesis and strategies for CRC prevention, with emphasis on risk stratification, optimization of endoscopic surveillance, dysplasia management, treat-to-target strategies and multidisciplinary management.

## Simple Summary

Patients with inflammatory bowel disease have an increased risk of developing colorectal cancer, particularly when inflammation affects the colon for many years. Preventing cancer in these patients requires more than regular colonoscopy. It also depends on identifying individual risk factors, detecting precancerous changes at an early stage, treating dysplasia appropriately, and maintaining good control of intestinal inflammation over time. This review summarizes the main mechanisms linking chronic intestinal inflammation to colorectal cancer and discusses current strategies for cancer prevention, including risk-based surveillance, advanced endoscopic techniques, management of dysplasia, and long-term disease control. A better integration of these approaches may help clinicians tailor prevention strategies to each patient and improve early detection while avoiding unnecessary procedures.

## 1. Introduction

Inflammatory bowel diseases (IBDs), including ulcerative colitis (UC) and Crohn’s disease (CD), are chronic, immune-mediated disorders characterized by relapsing–remitting inflammation of the gastrointestinal tract [1,2,3]. CRC represents a major long-term complication of IBD and remains a major contributor to disease-related morbidity and mortality [1,4]. Patients with extensive and long-standing UC (excluding isolated proctitis) and CD have an approximately two- to three-fold increased relative risk of CRC compared with the general population, although absolute risk estimates vary according to study design, calendar period, geographical region, and individual risk modifiers [1,2,5]. CRC risk increases with disease duration and becomes clinically relevant after approximately eight years from disease onset [2,5]. Additional determinants of CRC risk include cumulative inflammatory burden, extent and severity of disease, early age at IBD diagnosis, family history of CRC, and concomitant primary sclerosing cholangitis (PSC), while colonic strictures and post-inflammatory polyps are also incorporated into contemporary risk-assessment frameworks [1,2,6]. Persistent mucosal inflammation promotes oxidative stress, genomic damage, and aberrant epithelial regeneration, favoring the accumulation of genetic and epigenetic alterations and clonal expansion of premalignant cells [2,4]. In contrast to sporadic CRC, which typically arises through the conventional adenoma–carcinoma sequence, IBD-associated CRC develops within chronically inflamed mucosa (“field cancerization”) and follows a distinct dysplasia–carcinoma pathway [2,4]. Although the principal molecular pathways of sporadic CRC—chromosomal instability, microsatellite instability, and CpG island methylator phenotype—are also implicated, the temporal sequence of genetic events differs, with early TP53 alterations and relatively delayed APC mutations representing hallmarks of inflammation-associated tumorigenesis [2,4].

Population-based data indicate a decline in CRC incidence among patients with IBD over recent decades, which may reflect improvements in endoscopic surveillance, enhanced dysplasia detection through high-definition imaging and chromoendoscopy, and more effective long-term control of mucosal inflammation [1,2,5]. Nevertheless, CRC incidence and mortality remain higher than in the general population, and IBD-associated CRC continues to display less favorable outcomes, partly due to younger age at diagnosis, multifocal disease, and adverse histopathological features [2,5].

Consequently, CRC risk management in IBD has evolved from a surveillance-centered model to an integrated prevention strategy combining individualized risk stratification, high-quality endoscopic surveillance, timely dysplasia management, and sustained suppression of intestinal inflammation [1,2,5]. Treat-to-target disease management may complement surveillance by limiting cumulative inflammatory exposure, although its direct effect on CRC incidence remains to be established [5].

Despite international guidelines and consensus statements, substantial variability persists in surveillance intervals, endoscopic techniques, and preventive therapeutic strategies. Ongoing controversies include the chemopreventive role of 5-aminosalicylates, the long-term oncologic impact of immunomodulators and biologics, and the optimal management of invisible or low-grade dysplasia [1,2,5]. This review therefore aims to provide a critical synthesis of the current evidence on the prevention of CRC in patients with IBD, incorporating risk stratification, endoscopic surveillance, the management of dysplasia and the control of inflammation, with a view to supporting personalised preventive strategies.

## 2. Materials and Methods

A comprehensive literature search was conducted using the PubMed database to identify relevant studies addressing CRC risk, surveillance, and prevention in IBD. The search was performed up to August 2026. The search strategy included combinations of the following keywords: “colorectal cancer”, “CRC”, “colorectal neoplasia”, “malignancy”, “inflammatory bowel disease”, “IBD”, “ulcerative colitis”, “UC”, “Crohn’s disease”, “CD”, “surveillance”, “screening”, “risk stratification”, “dysplasia”, “chromoendoscopy”, and “inflammation”. Boolean operators (AND, OR) were applied to optimize the search strategy. The search was restricted to articles published in English. Original studies, systematic reviews, meta-analyses, and relevant clinical guidelines were considered eligible for inclusion.

## 3. Mechanisms of Colorectal Carcinogenesis in Inflammatory Bowel Disease: Clinical Implications

IBD-associated colorectal carcinogenesis is driven by chronic inflammation and follows biological trajectories that differ from those observed in sporadic CRC. Neoplastic transformation occurs within a persistently inflamed mucosal field and is frequently characterized by multifocality, flat dysplasia, and accelerated progression [4]. Sustained immune activation, epithelial barrier dysfunction, oxidative DNA damage, and microbial dysbiosis converge to promote genomic instability and clonal expansion of premalignant epithelial cells [4,7,8]. These interconnected processes establish a protumorigenic microenvironment that underlies the dysplasia–carcinoma sequence in IBD.

### 3.1. Inflammation-Driven Carcinogenesis in IBD

Chronic mucosal inflammation is the central driver of carcinogenesis in colonic IBD. Persistent inflammatory activity promotes the production of reactive oxygen species (ROS) and reactive nitrogen intermediates, leading to oxidative stress, DNA strand breaks, and accumulation of mutational events within epithelial stem cell compartments [4,9]. Recurrent cycles of mucosal injury and repair promote dysregulated epithelial proliferation, impaired apoptosis, and clonal expansion of genetically altered cell populations, thereby facilitating neoplastic progression [7,9]. These processes occur within a pro-tumorigenic microenvironment characterized by aberrant cytokine signaling, immune cell infiltration, and altered interactions among epithelial cells, stromal components, and the intestinal microbiota [4,7]. Several inflammatory pathways have been implicated in IBD-associated tumorigenesis. Activation of NF-κB and STAT3 signaling cascades plays a prominent role by sustaining pro-survival transcriptional programs, enhancing epithelial proliferation and promoting resistance to apoptosis [8,9]. Pro-inflammatory cytokines such as tumor necrosis factor (TNF), interleukin (IL)-6, and IL-23 contribute to a tumor-permissive microenvironment by sustaining chronic immune activation and epithelial regeneration in the setting of genomic instability [7,9]. Chronic inflammation may also impair immune surveillance, facilitating the persistence and expansion of dysplastic clones during early neoplastic evolution [7]. Beyond immune-mediated pathways, epithelial barrier dysfunction and microbial dysbiosis further contribute to inflammation-associated carcinogenesis. Disruption of mucosal integrity and alterations in the gut microbiome facilitate bacterial translocation and persistent antigenic stimulation, amplifying inflammatory signaling and increasing exposure to microbial-derived genotoxic metabolites [8,9]. Specific microbial taxa have been implicated in CRC development through the production of genotoxic metabolites, modulation of bile acid metabolism, and promotion of inflammatory signaling. In the setting of chronic colitis, microbial factors may further amplify epithelial stress responses and contribute to mutational selection within the inflamed mucosa [8,9].

### 3.2. Dysplasia–Carcinoma Sequence and Field Cancerization in IBD

IBD-associated CRC develops through a dysplasia–carcinoma pathway distinct from sporadic tumorigenesis. Whereas sporadic CRC typically follows the adenoma–carcinoma sequence originating from discrete polypoid lesions, IBD-associated neoplasia arises within chronically inflamed mucosa and may be multifocal, with synchronous or metachronous dysplastic lesions occurring in different colonic segments [4,7]. This pattern reflects the concept of field cancerization, whereby prolonged inflammatory exposure leads to widespread molecular alterations across the colonic epithelium, creating a permissive environment for clonal expansion and malignant transformation [4,7]. At the molecular level, IBD-associated dysplasia displays a distinct sequence of driver alterations. TP53 mutations occur early and can be detected even in non-dysplastic inflamed mucosa, suggesting a potential initiating role in inflammation-associated carcinogenesis [4,10]. In contrast, APC mutations—typically early events in sporadic CRC—tend to occur later and less consistently in colitis-associated cancer, supporting divergent oncogenic trajectories [4,10,11]. This altered molecular pattern may contribute to the development of flat or subtle lesions that are challenging to detect endoscopically. Genomic studies further support these mechanistic differences. Chatila et al. performed an integrated clinical and genomic analysis of colitis-associated cancers and identified distinct driver events and evolutionary patterns compared with sporadic CRC, including early TP53 alterations and evidence of convergent evolution among spatially separated dysplastic lesions [10]. Hirsch et al. reported that UC-associated CRCs display a genomic landscape characterized by frequent TP53 alterations and chromosomal aneuploidies, with TP53 representing the most commonly mutated gene in their cohort. Notably, spatially separated tumor lesions within the same patient often harbored distinct TP53 mutations, supporting the concept of independent clonal evolution within chronically inflamed mucosa [11].

**Figure 1:**
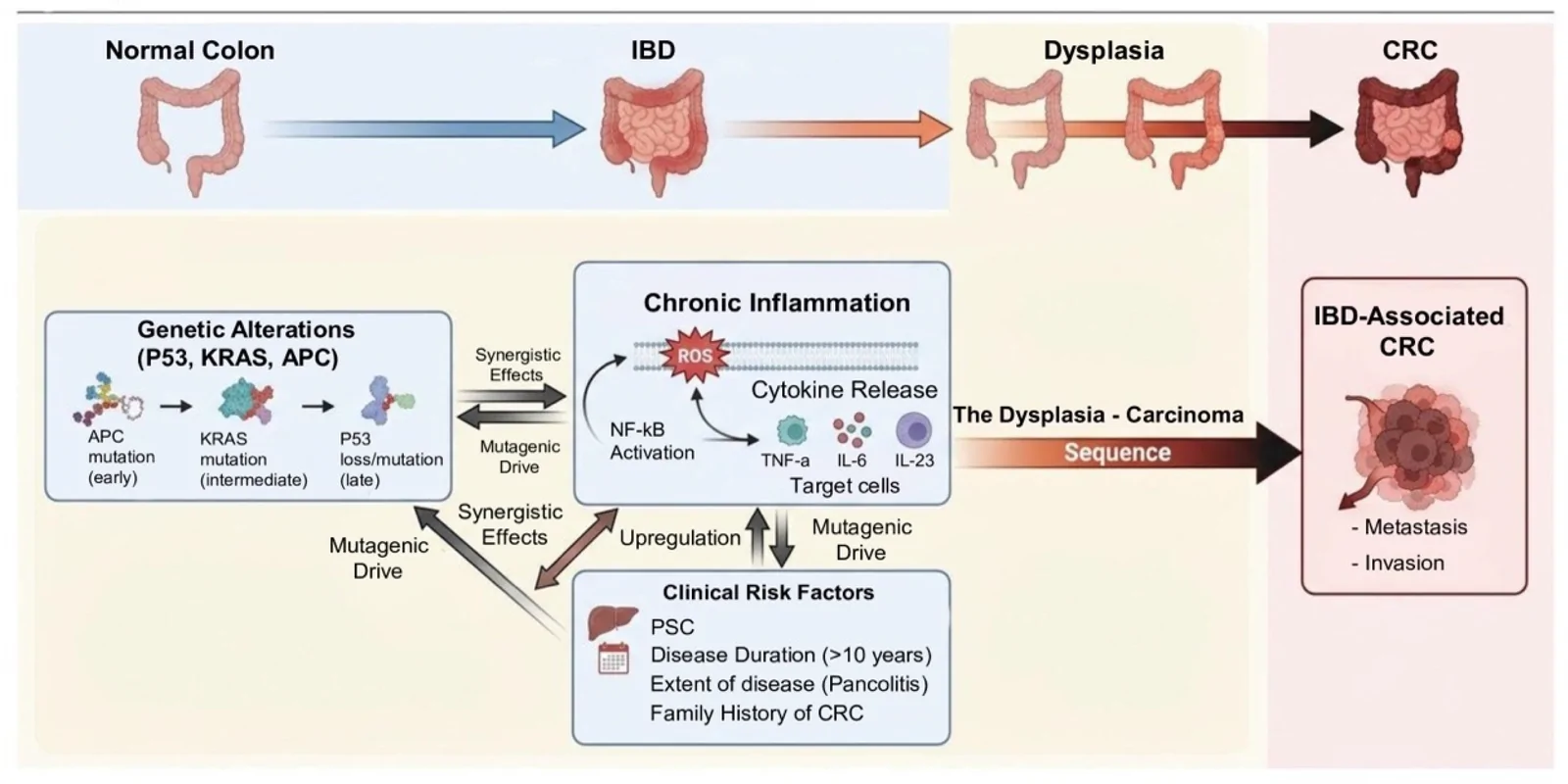
Schematic representation of CRC development in IBD. The figure highlights the interplay between chronic inflammation (oxidative stress, cytokine release), genetic alterations (TP53, KRAS, APC) and clinical risk factors (PSC, disease duration) leading to the dysplasia—carcinoma sequence. **IBD:** Inflammatory Bowel Disease; **CRC:** Colorectal Cancer; **PSC:** Primary Sclerosing Cholangitis; **ROS:** Reactive Oxygen Species. Figure created by the authors using Adobe Photoshop 2025 (Adobe Inc., San Jose, CA, USA).

Meta-analytic evidence further supports the central role of TP53 in colitis-associated tumorigenesis. In a meta-analysis including 19 studies, Du et al. found that TP53 mutations were significantly more frequent in IBD-associated CRC than in sporadic CRC, whereas KRAS mutations occurred less frequently. Both TP53 and KRAS alterations were more prevalent in IBD-CRC compared with IBD without dysplasia, highlighting their potential relevance as molecular markers of neoplastic progression in IBD [12]. From a clinical perspective, in a multicenter cohort analysis, Lightner et al. demonstrated similar rates of progression from low- or high-grade dysplasia to CRC in patients with UC and Crohn’s colitis, supporting similar principles of dysplasia surveillance and management across colonic IBD phenotypes [13]. The main inflammatory, molecular, and clinical pathways involved in IBD-associated colorectal carcinogenesis are summarized in Figure 1.

### 3.3. Why These Mechanisms Matter for Prevention: Clinical Implications

The molecular basis of IBD-associated CRC has important implications for prevention. The central role of chronic inflammation provides the biological rationale for treat-to-target strategies aimed at achieving durable mucosal healing, while the multifocal nature of colitis-associated neoplasia highlights the importance of effective endoscopic surveillance [4,7,8,9,10,11]. Furthermore, the specific molecular profile of carcinogenesis associated with IBD could provide a basis for future translational approaches, although these are still at an experimental stage [10,11,12].

## 4. Risk Stratification

Effective prevention of CRC in IBD relies on the early identification of patients at increased risk. CRC risk in IBD is heterogeneous and is influenced by multiple patient-, disease-, and neoplasia-related factors [2,3]. Contemporary risk stratification frameworks integrate these determinants into clinically actionable categories that guide surveillance intensity [14].

### 4.1. Disease Duration, Extent, and Cumulative Inflammatory Burden

Disease duration remains one of the most consistently validated predictors of CRC risk in colonic IBD. Early epidemiological studies demonstrated a progressive increase in CRC incidence with longer-standing colitis, providing the basis for time-dependent surveillance strategies. In a landmark meta-analysis including 116 studies and nearly 54,000 patients with UC Eaden et al. reported a cumulative CRC risk of approximately 2% after 10 years of disease, 8% after 20 years, and 18% after 30 years of colitis duration, with an overall CRC prevalence of 3.7% among UC patients and an even higher prevalence in those with extensive colitis [6]. These historical estimates should be interpreted in the context of contemporary cohorts reporting substantially lower absolute risks. Nevertheless, disease duration remains an established component of contemporary risk assessment [2,3,15].

The extent of colonic involvement represents an additional major determinant. Extensive colitis has consistently been associated with a higher CRC risk compared with limited disease and disease extent remains incorporated into contemporary risk-stratification frameworks. Beyond duration and extent, cumulative inflammatory burden has emerged as a central driver of CRC risk. Persistent mucosal inflammation promotes oxidative stress, DNA damage, and dysregulated epithelial regeneration, accelerating neoplastic progression along the dysplasia–carcinoma pathway [2,3]. These observations underscore that CRC risk in IBD is dynamic and evolves over time in relation to disease control, rather than representing a static attribute based solely on historical disease characteristics [2,3].

### 4.2. Major Risk Modifiers

Several additional factors further modify CRC risk and should be systematically incorporated into risk stratification and surveillance planning.

PSC is among the strongest independent risk factors for CRC in IBD. Patients with PSC-associated colitis have a markedly increased CRC risk compared with IBD patients without PSC, and are consistently classified as a high-risk subgroup requiring annual surveillance [2,3].

A family history of CRC in a first-degree relative is associated with an almost eightfold higher CRC risk in patients with IBD (SIR 7.9; 95% CI 1.6–14.3), supporting its inclusion in risk stratification and surveillance planning [16].

A personal history of colonic dysplasia or strictures represents one of the strongest predictors of subsequent advanced neoplasia. Surveillance cohorts have reported hazard ratios for advanced colorectal neoplasia generally ranging from 3 to 5 in patients with prior dysplasia, underscoring its central role in both risk stratification and preventive decision-making [17]. In contrast, other historically proposed risk markers have not been confirmed as independent determinants of colorectal neoplasia. Inflammatory pseudopolyps, long considered indicators of increased CRC risk, have not been shown to independently predict neoplastic progression. A large multicenter cohort study demonstrated no significant association between the presence of pseudopolyps and the risk of colorectal neoplasia after adjustment for confounding factors, suggesting that pseudopolyps primarily reflect prior or cumulative inflammatory burden rather than a direct carcinogenic effect [18]. From a practical point of view, post-inflammatory polyps can therefore serve as an easily identifiable endoscopic indicator of a previous inflammatory burden, particularly when longitudinal endoscopic or histological data are incomplete. However, their presence or number should not be regarded as a validated quantitative measure of cumulative inflammation and should be interpreted in the light of longitudinal inflammatory activity, disease extent and other established risk factors [18].

### 4.3. Clinical Implementation and Limitations of Risk Stratification

Current risk stratification frameworks recommend intensified surveillance for high-risk patients, such as those with PSC, prior dysplasia, or persistent inflammation, while allowing longer surveillance intervals for low-risk individuals, reflecting the shift toward personalized, risk-adapted prevention strategies [14]. However, their clinical applicability remains limited by heterogeneity in study design, variability in the assessment of inflammatory activity, and differences in surveillance protocols across cohorts [2,3,17]. Furthermore, current models rely predominantly on clinical and endoscopic variables, as validated molecular biomarkers for individualized CRC risk prediction are not yet available for routine clinical practice [2,3]. In this context, molecular alterations such as those in the TP53 and KRAS genes could potentially complement established clinical and endoscopic risk factors, contributing to a more personalised identification of patients at higher risk of tumour progression [12].

## 5. Endoscopic Surveillance as the Cornerstone of CRC Prevention

Endoscopic surveillance represents the cornerstone of CRC prevention in patients with colonic IBD. Its principal aims are to detect dysplasia and early cancer at a curable stage while enabling endoscopic resection of visible precancerous lesions when feasible.

Advances in endoscopic imaging and lesion characterization have reshaped IBD surveillance, leading to contemporary recommendations emphasizing high-quality colonoscopy, risk-adapted surveillance, and advanced imaging techniques [19,20,21,22].

### 5.1. Timing and Risk-Adapted Surveillance Intervals

The timing of surveillance initiation is closely related to disease duration and extent, reflecting the progressive increase in CRC risk over time in patients with longstanding colitis. Current guidelines generally recommend an initial screening colonoscopy approximately eight years after the onset of IBD symptoms in patients with colonic involvement, with earlier initiation in high-risk scenarios such as PSC [20,21,22]. Surveillance intervals should be individualized according to patient risk profiles. Both ECCO and AGA recommendations support shorter intervals for patients with high-risk features and longer intervals for individuals with quiescent disease and no additional risk modifiers [20,21]. The British Society of Gastroenterology (BSG) guidelines have further refined these recommendations by incorporating cumulative inflammatory burden and endoscopic findings into structured risk categories, supporting increasingly personalized surveillance schedules [22]. Nevertheless, significant differences remain between international recommendations. The ECCO proposes relatively fixed intervals, stratified according to risk: 1 year for high-risk patients, 2–3 years for those at intermediate risk, and 5 years for patients without intermediate or high-risk characteristics [21]. In contrast, the AGA adopts a more flexible interval, ranging from 1 to 5 years, based on an individualised assessment of CRC risk factors and the quality of previous surveillance [20]. More recently, the BSG guidelines have moved towards a more personalised approach based on absolute cancer risk, allowing selected patients, whose risk is close to that of the general population, to undergo a repeat colonoscopy every 10 years, alongside population-based, age-appropriate CRC screening [22]. These discrepancies reflect the persistent uncertainty regarding the optimal intensity of surveillance in low-risk IBD populations and highlight the limited evidence supporting specific surveillance intervals. A related clinical question concerns the circumstances under which surveillance may reasonably be discontinued. The need for continued surveillance should be periodically reassessed, particularly in older patients and those with substantial comorbidity or limited life expectancy, considering recent colonoscopic findings, prior neoplasia, procedural risk, and patient preferences [23]. Although no specific age threshold has been established for discontinuing screening, the potential benefit of detecting asymptomatic cancers should be weighed against the risks associated with the procedure, comorbidities, frailty and life expectancy. In selected patients, where the associated risks outweigh the expected benefit of continuing surveillance, discontinuation may therefore be considered, following an individualised assessment and in accordance with a shared decision-making approach [22,23].

### 5.2. Advanced Endoscopic Imaging for Dysplasia Detection

Advances in endoscopic imaging have significantly improved dysplasia detection in IBD surveillance. The transition from standard-definition white-light endoscopy (WLE) to high-definition (HD) colonoscopy has enhanced mucosal visualization and lesion recognition, and HD imaging is now considered the preferred platform for surveillance procedures [24,25]. Dye-based chromoendoscopy (DCE), typically performed using indigo carmine or methylene blue, improves mucosal contrast and facilitates identification of subtle lesions. The SCENIC international consensus strongly endorsed chromoendoscopy with targeted biopsies as a preferred surveillance strategy, particularly in patients with long-standing colitis [19]. Prospective and comparative studies have demonstrated improved detection of dysplasia with chromoendoscopy compared with standard-definition WLE [24]. Virtual chromoendoscopy (VCE) techniques—including narrow-band imaging (NBI), i-scan and linked colour imaging—have established themselves as practical alternatives to dye-based methods. These technologies eliminate the need for dye application and simplify procedural workflows. Randomised studies have shown that VCE is at least as effective as DCE in detecting neoplasms [26]. The latest ECCO guidelines recognise high-definition VCE alongside DCE for the surveillance of IBD [27]. A recent network meta-analysis suggested that HD DCE may provide a modest advantage over HD-WLE for dysplasia detection, although the certainty of evidence was low, while evidence for other modality comparisons remained less robust [28]. Accordingly, the choice of imaging modality should also consider available expertise, equipment, and procedural quality. Importantly, surveillance effectiveness depends not only on imaging modality but also on careful mucosal inspection and endoscopist expertise. In a multicenter case–control study, surveillance compliant with predefined quality indicators was associated with a lower risk of colitis-associated advanced neoplasia [29].

The comparative diagnostic performance and main characteristics of the endoscopic imaging modalities currently used for dysplasia surveillance in IBD are summarized in Table 1.

### 5.3. Targeted Versus Random Biopsies

Historically, surveillance relied on random four-quadrant biopsies obtained every 10 cm throughout the colon to compensate for the presumed high prevalence of invisible dysplasia. However, this approach is labor-intensive and often yields limited diagnostic benefit in the era of high-definition imaging. The SCENIC consensus marked a paradigm shift by recommending targeted biopsies of visible lesions during chromoendoscopy as the preferred strategy [19]. Subsequent studies have confirmed that the majority of dysplasia is endoscopically visible when modern imaging techniques are used. For example, Watanabe et al., in a multicenter randomized controlled trial including 246 patients with long-standing UC, demonstrated that targeted biopsies during surveillance colonoscopy detected neoplasia at rates comparable to random four-quadrant biopsies (11.4% vs. 9.3%, respectively; *p* = 0.617), while requiring substantially fewer biopsy samples (3.1 vs. 34.8 per colonoscopy) and shorter examination times (26.6 vs. 41.7 min; *p* < 0.001), supporting the efficiency of targeted surveillance strategies in the era of high-definition endoscopy [31]. Consequently, current guidelines recommend performing targeted biopsies during high-definition chromoendoscopy, whilst in selected high-risk patients—particularly those with PSC, a history of colon cancer or dysplasia, strictures, or persistent inflammation refractory to treatment—additional non-targeted biopsies may be considered in the four quadrants at 10 cm intervals [20,21,22,27].

### 5.4. Surveillance Quality and Clinical Outcomes

High-quality colonoscopy is increasingly recognized as a key determinant of surveillance effectiveness. Variability in dysplasia detection may reflect not only patient-related risk factors but also procedural quality, endoscopist expertise, and technical performance. Adequate bowel preparation, meticulous mucosal inspection, and systematic documentation are therefore essential components of effective surveillance programs [32]. Additional considerations in IBD surveillance include accurate assessment of disease extent, recognition of post-inflammatory pseudopolyps, and evaluation of strictures. Active inflammation itself represents an important risk factor for neoplasia; observational data from Rutter et al. demonstrated that increasing inflammatory severity is associated with a higher risk of colorectal neoplasia [33]. Close collaboration with expert gastrointestinal pathologists is also essential, as dysplasia grading may be subject to interobserver variability and accurate histologic interpretation is critical for guiding management decisions. Although randomized trials assessing CRC mortality reduction through surveillance are limited, observational studies support the clinical benefit of structured surveillance programs. Choi et al. reported that surveillance colonoscopy was associated with reduced CRC-related mortality in UC, consistent with earlier-stage cancer detection and improved opportunities for curative intervention [34].

## 6. Management of Dysplasia to Prevent Colorectal Cancer

The detection of dysplasia during surveillance colonoscopy represents a critical event in the management of IBD, as it identifies patients at increased risk of synchronous or metachronous colorectal neoplasia and requires prompt implementation of appropriate preventive strategies. Historically, diagnosis of dysplasia in UC or CD colitis frequently led to colectomy because of concerns regarding synchronous and metachronous neoplastic lesions and the limited sensitivity of conventional WLE. However, advances in high-definition imaging, chromoendoscopy, and endoscopic resection techniques have substantially modified this paradigm. Current evidence and international guidelines increasingly support organ-preserving strategies based on careful lesion characterization and complete endoscopic resection when feasible, followed by intensified surveillance and risk-adapted follow-up [19,20,21,22]. In this context, dysplasia management requires integration of several key factors, including dysplasia visibility (visible vs. invisible), histological grade (LGD vs. HGD), focality (unifocal vs. multifocal), endoscopic resectability, and patient-related risk modifiers such as PSC, extent of colitis, cumulative inflammatory burden, and prior dysplasia history [20,21,22]. Current recommendations also emphasize that complex dysplasia management should ideally be performed in specialized centers with expertise in IBD surveillance and advanced therapeutic endoscopy [20,21,22]. Consequently, clinical management should be based on the distinction between visible and invisible dysplasia, the histological grade, the focal nature of the lesion and its endoscopic resectability; endoscopic resection is preferable for visible lesions that are completely resectable and lack features indicative of invasive carcinoma, whilst surgical management should be considered when complete endoscopic resection is not feasible or when high-risk features are present, such as persistent invisible HGD or multifocal dysplasia [19,20,21,22].

### 6.1. Dysplasia Classification and Clinical Relevance

Dysplasia in IBD is primarily classified according to its endoscopic visibility, distinguishing visible dysplasia from invisible dysplasia detected on non-targeted biopsies. This distinction has direct implications for clinical management and is strongly emphasized in contemporary consensus statements [19,20,22]. Histological grading also remains essential, as the risk of progression to CRC increases from LGD to HGD, with higher-grade lesions being associated with a greater likelihood of synchronous malignancy.

However, histological diagnosis of dysplasia is limited by significant interobserver variability, particularly for LGD and “indefinite for dysplasia”. For this reason, expert gastrointestinal pathology review is recommended before definitive management decisions are made. This approach is particularly important in cases of invisible dysplasia, where clinical decisions may rely entirely on histological interpretation in the absence of a visible lesion [20,35].

### 6.2. Management of Visible Dysplasia

#### 6.2.1. Endoscopic Resection as First-Line Strategy

Visible dysplasia should generally be managed with endoscopic resection when complete excision is technically feasible and invasive carcinoma is not suspected. The SCENIC consensus and subsequent international guidelines recommend chromoendoscopy-guided evaluation with targeted resection as the preferred strategy [19,20,21,22]. When complete endoscopic removal is achieved, colectomy is not routinely required and patients can undergo close surveillance. Lesions should be carefully assessed based on morphology, surface pattern, and features suggestive of submucosal invasion, as suspected invasive disease should prompt surgical evaluation [20,21,22].

#### 6.2.2. EMR Versus ESD in Colitis-Associated Neoplasia

**Figure 2:**
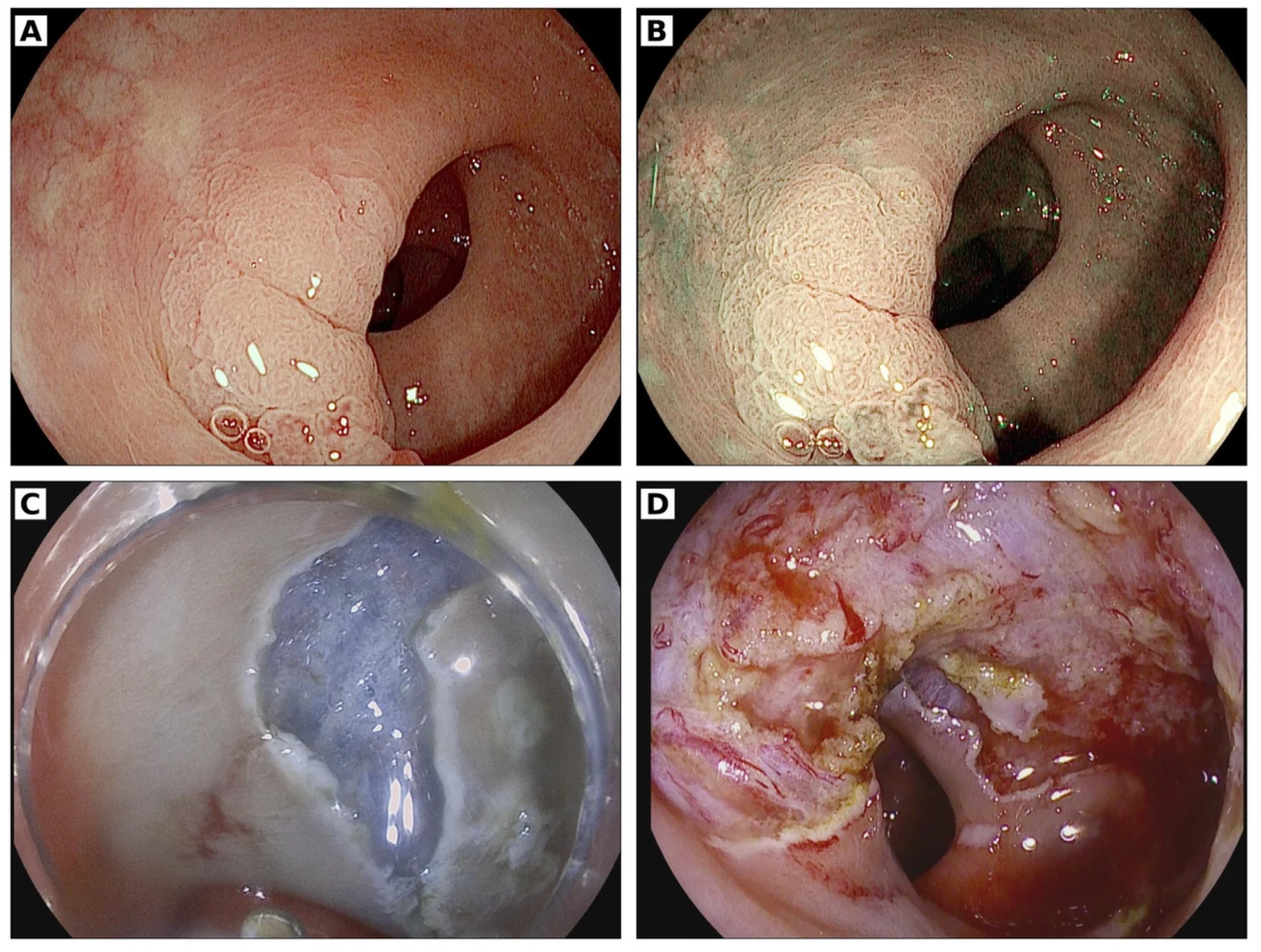
Endoscopic assessment and treatment of a visible sigmoid lesion in ulcerative colitis. (**A**) White-light endoscopic view of the visible sigmoid lesion. (**B**) Virtual chromoendoscopy with iScan, providing enhanced visualization of the lesion surface and margins. (**C**) Endoscopic submucosal dissection (ESD) during the dissection phase. (**D**) Post-ESD mucosal defect after complete endoscopic resection. Endoscopic images provided by the Gastroenterology and Digestive Endoscopy Unit, IRCCS San Raffaele Scientific Institute, Milan, Italy.

The choice of resection technique depends on lesion characteristics and the presence of fibrosis, which is common in chronically inflamed mucosa. Endoscopic mucosal resection (EMR) is typically used for well-demarcated lesions amenable to en bloc or, when appropriate, piecemeal excision, whereas endoscopic submucosal dissection (ESD) may be preferred for larger lesions, non-lifting lesions, or when en bloc resection is required for accurate histological assessment. ESGE guidelines recognize ESD as an important technique for superficial gastrointestinal neoplasia, particularly in expert centers, due to its higher rates of complete (R0) resection [36]. Emerging evidence in IBD-associated dysplasia also supports the feasibility of ESD when performed by experienced endoscopists [37,38]. An example of multimodal endoscopic assessment and ESD treatment of a visible colonic lesion in ulcerative colitis is shown in Figure 2.

#### 6.2.3. Surveillance After Complete Resection

Even after complete endoscopic resection of visible dysplasia, patients remain at risk of metachronous neoplasia due to the underlying field effect. Therefore, post-resection surveillance should be tailored to lesion- and patient-related risk features, with early reassessment within 3–6 months particularly appropriate for higher-risk lesions, followed by risk-adapted surveillance [39,40].

### 6.3. Management of Invisible Dysplasia

Invisible dysplasia represents one of the most challenging scenarios in IBD-related cancer prevention. Dysplasia detected on random biopsies in the absence of a visible lesion raises concern for missed flat dysplasia or multifocal neoplasia. Current recommendations therefore emphasize a stepwise approach, including confirmation of the diagnosis by an expert gastrointestinal pathologist and repeat colonoscopy performed under optimal conditions using high-definition chromoendoscopy by an experienced endoscopist [19,20,22,35]. If a visible lesion is subsequently identified, endoscopic resection should be attempted whenever technically feasible.

#### 6.3.1. Invisible Low-Grade Dysplasia

The management of confirmed invisible LGD remains controversial and requires careful risk stratification. Evidence from meta-analyses and long-term cohort studies indicates that patients with LGD have a measurable risk of progression to advanced neoplasia or CRC over time, particularly in the presence of multifocal dysplasia or additional risk factors such as PSC or extensive colitis [41,42].

After confirmation of LGD, repeat high-quality chromoendoscopy is recommended to exclude a previously undetected lesion. If no visible dysplasia is identified, close surveillance may be considered in selected patients. However, persistent invisible LGD despite optimized endoscopic evaluation raises concern for occult neoplasia and may justify colectomy, particularly in patients with additional high-risk features [20,21,22]. The choice between ongoing surveillance and surgical treatment should be tailored to the extent and location of the dysplasia and the presence of additional risk factors, such as PSC or extensive colitis. In the absence of additional high-risk features, non-visible LGD does not necessarily require colectomy and can be managed with careful, risk-adjusted surveillance following an appropriate endoscopic re-evaluation [20,21,22,41,42].

#### 6.3.2. Invisible High-Grade Dysplasia and Multifocal Dysplasia

Invisible HGD represents a substantially higher-risk condition due to the increased likelihood of synchronous advanced neoplasia and the limitations of endoscopic diagnosis, even with optimised imaging techniques. When HGD is confirmed by a pathologist specialising in gastrointestinal pathology, a new, high-quality chromoendoscopy must be performed to rule out the presence of a previously unrecognised visible lesion. If no resectable lesion is identified and the invisible HGD persists, colectomy must be seriously considered, particularly in the presence of additional high-risk features, with a final, individualised decision based on lesion characteristics and patient factors and made following a multidisciplinary assessment [20,21,22].

### 6.4. Surgical Management and Oncologic Considerations

Despite the increasing role of advanced endoscopic resection techniques, surgery remains a critical component of dysplasia management in IBD. Surgical referral is generally recommended when dysplasia cannot be completely resected endoscopically, when invasive carcinoma is suspected, or when dysplasia characteristics suggest a high risk of synchronous or metachronous malignancy, such as persistent invisible HGD or multifocal dysplasia [20,21,22].

Current surgical guidelines emphasize individualized decision making based on disease distribution, patient comorbidities, functional status, and quality-of-life considerations, particularly when colectomy is performed primarily for cancer prevention rather than for medically refractory disease [43].

Patient age and competing mortality risks should also be considered when evaluating surgical indications. In elderly patients or individuals with significant comorbidities, the potential oncologic benefit of colectomy may be outweighed by operative risk, requiring a personalized approach that integrates life expectancy and patient preferences into the decision-making process [23].

### 6.5. Multidisciplinary Decision Making and Long-Term Follow-Up

**Figure 3:**
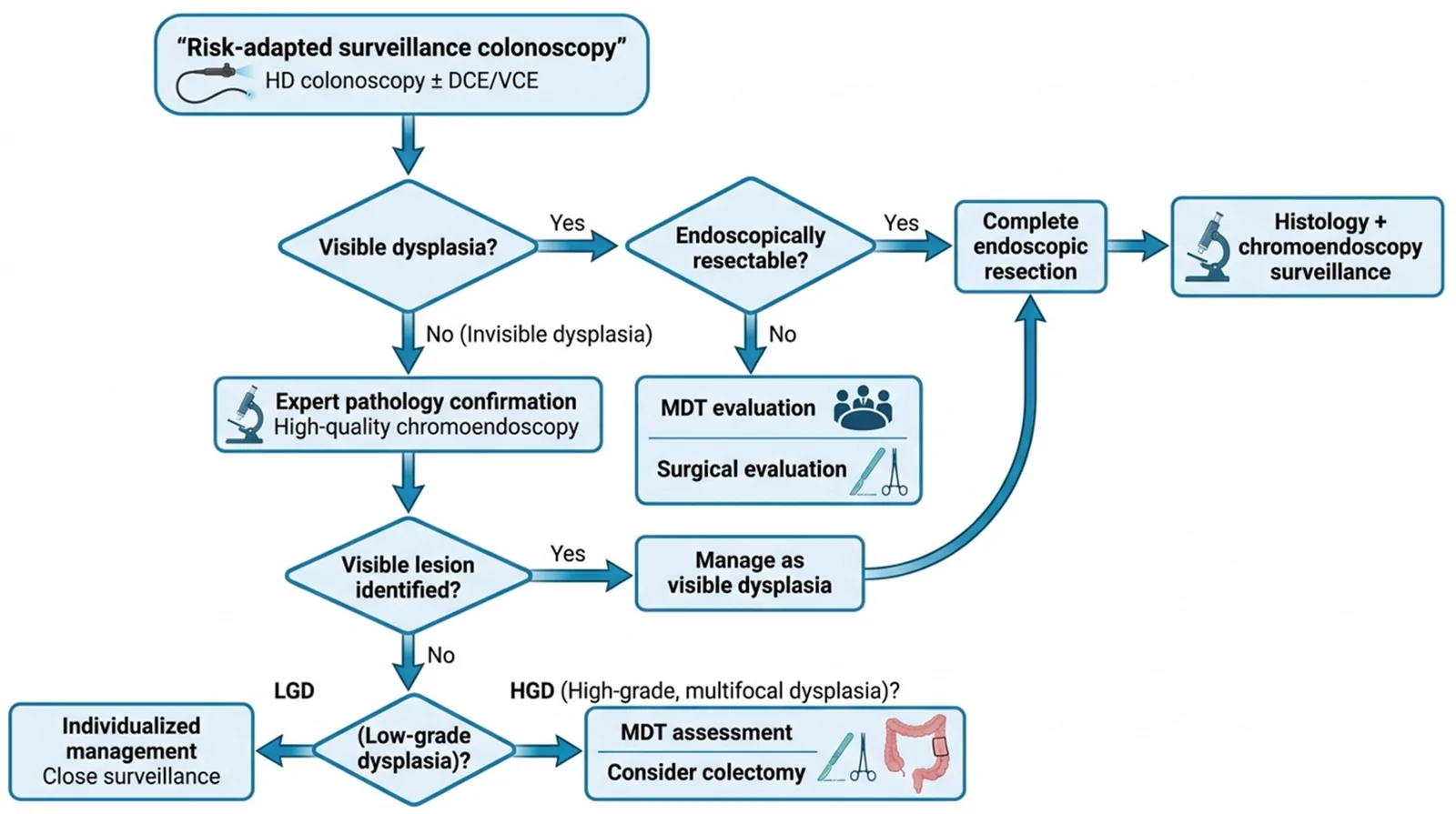
Practical management pathway for dysplasia in colonic inflammatory bowel disease. The algorithm summarizes risk-adapted surveillance, management of visible and invisible dysplasia, assessment of endoscopic resectability, and indications for intensified surveillance or surgical evaluation. DCE, dye-based chromoendoscopy; HD, high-definition; HGD, high-grade dysplasia; IBD, inflammatory bowel disease; LGD, low-grade dysplasia; MDT, multidisciplinary team; VCE, virtual chromoendoscopy. Figure created by the authors using Figures (Figures Labs, Inc., San Francisco, CA, USA).

Given the heterogeneity of dysplasia presentations and the potential consequences of surgical intervention, multidisciplinary decision making has become central to the management of complex dysplasia in IBD. Invisible dysplasia, multifocal LGD, lesions requiring advanced resection techniques, or patients with elevated operative risk may benefit from coordinated evaluation by IBD specialists, advanced endoscopists, expert gastrointestinal pathologists, and colorectal surgeons [20,22,39,43]. A practical algorithm summarizing the management of dysplasia in colonic IBD, including surveillance, endoscopic resection, and indications for surgical evaluation, is shown in Figure 3.

## 7. Inflammation Control as an Indirect Strategy to Reduce CRC Risk

The central role of chronic inflammation in IBD-associated carcinogenesis supports the integration of sustained disease control into modern CRC prevention strategies. Contemporary preventive strategies therefore extend beyond early detection and include therapeutic approaches aimed at achieving sustained mucosal healing and long-term control of the disease [44,45].

### 7.1. Impact of Persistent Inflammation on CRC Risk

The association between chronic inflammation and CRC risk in IBD has been consistently demonstrated across multiple cohort studies. Histologic and endoscopic inflammatory activity represent independent predictors of colorectal neoplasia rather than simple markers of disease severity. Gupta et al. showed that greater histologic inflammatory activity over time was independently associated with progression to advanced colorectal neoplasia in UC [46].

More recently, the concept of cumulative inflammatory burden has provided a more refined model for CRC risk prediction. In a landmark study, Choi et al. demonstrated that longitudinal exposure to inflammation predicts colorectal neoplasia more accurately than single time-point assessments, highlighting that cancer risk reflects the cumulative trajectory of disease activity [44]. These observations support the concept that CRC risk in IBD is dynamic and closely linked to sustained inflammatory exposure.

Together, mechanistic and epidemiological evidence provides a strong rationale for sustained inflammation control as a component of CRC-risk reduction [44,46,47,48].

### 7.2. Treat-to-Target Strategies and Cancer Prevention Through Remission

The treat-to-target paradigm has shifted IBD management from symptom control toward objective disease remission, with potential implications for long-term CRC prevention.

Evidence indicates that CRC risk correlates strongly with cumulative inflammatory exposure, suggesting that sustained disease control may reduce the likelihood of dysplasia development [44,46]. Accordingly, durable remission provides a biologically plausible means of reducing long-term inflammation-driven carcinogenic risk. In a retrospective multicenter cohort study of 5239 IBD patients followed for 19,820 patient-years within a treat-to-target framework, Parigi et al. reported 71 malignancies in 70 patients, corresponding to an overall incidence of 358 cases per 100,000 patient-years and a standardized incidence ratio (SIR) of 0.93 (95% CI 0.73–1.16) compared with the general population. CRC occurred in nine patients, with an incidence of 45 per 100,000 patient-years and an SIR of 1.18 (95% CI 0.54–2.09). Among malignancies detected within structured surveillance programs, early-stage disease (stage I–II) was significantly more frequent than advanced-stage disease (20 vs. 4 cases; *p* < 0.05) [5]. Although these observational data do not establish a direct chemopreventive effect of treat-to-target management, they support the integration of tight disease control and regular surveillance into long-term IBD care [5]. The expert literature further emphasizes that chronic inflammation represents a major modifiable determinant of CRC risk in IBD and that modern therapeutic approaches may indirectly contribute to risk reduction by achieving deep and sustained remission [49].

### 7.3. Medical Therapies and Evidence on CRC Risk Modulation

Medical therapy may influence CRC risk in IBD primarily through sustained control of mucosal inflammation. It is important to emphasise that the available data linking individual medical treatments to the risk of CRC are derived predominantly from observational studies and should therefore be interpreted as an association rather than as evidence of a causal chemopreventive effect. In UC, 5-Aminosalicylates (5-ASA) have long been considered potential chemopreventive agents, and a comprehensive systematic review and meta-analysis by Bonovas et al. suggested an association between 5-ASA use and a reduced risk of colorectal neoplasia, particularly in UC, although heterogeneity and methodological limitations preclude firm causal conclusions [50]. Current ECCO guidelines recommend 5-ASA for maintenance of remission in mild-to-moderate UC, while suggesting its discontinuation in patients initiating or achieving remission with advanced therapies or immunomodulators, as its chemopreventive role remains uncertain [51].

Thiopurines (azathioprine and 6-mercaptopurine) have been investigated for potential chemopreventive effects in IBD. However, a meta-analysis of 15 observational studies found no significant overall reduction in colorectal neoplasia risk (OR 0.87; 95% CI 0.71–1.06). Although a non-significant protective trend was observed when dysplasia and CRC were analyzed together (OR 0.72; 95% CI 0.50–1.05), no reduction in CRC risk alone was demonstrated (OR 0.90; 95% CI 0.72–1.12) [52]. Conversely, some observational cohorts have reported a protective association. In the ENEIDA registry, including 831 patients with UC, thiopurine exposure was independently associated with a reduced risk of advanced neoplasia (OR 0.21; 95% CI 0.06–0.74; *p* = 0.015) [53]. Thus, although some observational data suggest a potential protective association, a direct chemopreventive effect of thiopurines remains uncertain. In addition, thiopurines are associated with an increased risk of non-melanoma skin cancer, highlighting the need for careful risk–benefit assessment [54].

Anti-TNF therapy may potentially modulate CRC risk through sustained control of intestinal inflammation. Observational evidence remains inconsistent: a large multicenter database study reported a lower likelihood of CRC among anti-TNF-treated patients with both UC and CD, whereas a nationwide French cohort found a significant risk reduction only in patients with long-standing UC. Moreover, a meta-analysis did not demonstrate a significant overall protective association with advanced colorectal neoplasia. Therefore, current evidence does not establish a direct chemopreventive effect of anti-TNF therapy [17,55,56]. For newer advanced therapies, evidence regarding a direct effect on CRC risk remains limited. Available studies have mainly focused on overall malignancy risk, and available safety data should not be interpreted as evidence of CRC chemoprevention. Strategies targeting the microbiota, in particular faecal microbiota transplantation (FMT), have also been investigated as potential approaches to modulate gut dysbiosis and inflammatory activity in IBD. Recent ECCO guidelines recognise FMT as a promising approach in UC, with short-term studies showing higher rates of clinical and endoscopic remission compared with controls, although the certainty of the evidence remains limited and it is not currently possible to make any formal recommendations for its routine use [51]. Consistent with the above, the AGA advises against the use of conventional FMT for the treatment of IBD outside the context of clinical trials [57]. It is not yet known whether FMT-induced modulation of the gut microbiota translates into a reduction in colorectal neoplasia or the risk of CRC, and this requires specific long-term studies.

## 8. Special High-Risk Clinical Scenarios

Certain clinical scenarios confer a particularly high risk of CRC in IBD and require tailored surveillance and preventive strategies. These include PSC-associated IBD, patients with previous dysplasia or CRC, and elderly individuals with long-standing colitis.

### 8.1. Primary Sclerosing Cholangitis-Associated IBD

A meta-analysis including 64 studies and 776,700 patients with IBD reported a pooled PSC prevalence of 2.16%, with higher rates in UC than in CD (2.47% vs. 0.96%, respectively). Concomitant PSC identifies one of the highest-risk phenotypes for colorectal neoplasia in IBD and warrants a dedicated surveillance strategy [21,22,58]. Compared with non-PSC IBD, PSC-associated colitis is characterized by a markedly increased risk of colorectal neoplasia that is not fully explained by disease duration or inflammatory activity alone [59]. In a recent Swedish nationwide cohort, Abdalla et al. reported that CRC risk was particularly pronounced in younger patients with PSC-IBD; among patients diagnosed with IBD at ≤20 years of age, concomitant PSC was associated with an incidence rate ratio of 74.97 (95% CI 44.7–126.1) compared with population controls. PSC-associated CRC also showed a predominantly proximal distribution, with 37.5% of cancers located in the cecum/ascending colon and 23.4% in the transverse colon, and synchronous CRC was more frequent in PSC-positive patients than in population controls (4.7% vs. 1.9%) [60].

Reflecting this high-risk profile, current guidelines recommend annual colonoscopic surveillance starting at the time of PSC diagnosis, irrespective of colitis duration or activity [22]. Surveillance should ideally be performed using high-definition colonoscopy with chromoendoscopy and targeted biopsies, with consideration of additional random biopsies due to the higher diagnostic yield for invisible dysplasia in high-risk patients [22,59].

### 8.2. Patients with Previous Dysplasia or Colorectal Cancer

A prior history of colonic dysplasia represents one of the strongest predictors of subsequent advanced neoplasia in IBD. A systematic review and meta-analysis by Wijnands et al. confirmed that previous dysplasia is among the most consistent predictors of advanced colorectal neoplasia in surveillance cohorts, supporting the classification of these patients as a high-risk group requiring intensified follow-up [17]. In the era of high-definition colonoscopy and chromoendoscopy, visible dysplastic lesions can frequently be managed with complete endoscopic resection followed by intensified surveillance, allowing colectomy to be avoided in selected patients without features suggestive of invasive cancer or multifocal disease [61]. Patients with prior CRC represent an even higher-risk subgroup. When residual colonic mucosa remains after cancer treatment, management requires individualized surveillance and multidisciplinary evaluation to determine whether intensified endoscopic follow-up is sufficient or whether surgical intervention should be considered [3,61].

### 8.3. Elderly Patients with Long-Standing IBD: Balancing Surveillance Benefit and Competing Risks

The increasing prevalence of elderly individuals with long-standing IBD has introduced new challenges in CRC prevention [62]. Axelrad and Cross proposed a pragmatic framework for surveillance cessation in IBD, suggesting that discontinuation may be appropriate in selected patients with limited life expectancy or substantial procedural risk, particularly in the absence of high-risk features such as PSC or previous dysplasia [23]. The BSG guidelines also recommend periodic reassessment of the appropriateness of continued surveillance, incorporating age, comorbidity, life expectancy, previous neoplasia, and recent colonoscopic findings [22].

## 9. Integrated Strategies for CRC Prevention in IBD

CRC prevention in colonic IBD requires an integrated, risk-adapted approach. Disease activity should be monitored using objective measures and therapy optimized accordingly, while surveillance intensity should be periodically reassessed according to evolving oncologic risk. The key components of this integrated approach are summarized in Table 2 [27,45,63].

## 10. Conclusions and Future Perspectives

### 10.1. Summary of Current Prevention Strategies

The prevention of CRC in patients with IBD should be viewed as a dynamic, risk-adapted process, rather than a strategy based solely on surveillance. Current population-based data suggest a decline in the incidence of CRC compared with historical estimates, which may reflect improvements in surveillance, the detection of dysplasia and the control of inflammation [64].

Contemporary CRC prevention strategies combine individualized risk assessment, high-quality surveillance, dysplasia management, and sustained disease control. Advances in endoscopic imaging and resection increasingly allow organ-preserving treatment in selected patients, whereas those with high-risk features still require intensified, personalized prevention.

### 10.2. Unmet Needs and Future Directions in Personalized Prevention

There are still several challenges to be addressed in the prevention of CRC in patients with IBD. Current risk stratification models are based predominantly on clinical and endoscopic variables, whilst validated molecular biomarkers capable of accurately predicting progression to advanced cancer are not yet available for routine clinical practice [17].

Emerging technologies, including molecular endoscopy and artificial intelligence-assisted endoscopy, could improve the detection of dysplasia and the characterisation of lesions, but their role in the routine surveillance of IBD is still under investigation [65,66,67,68]. Similarly, the long-term oncological impact of the biologics and small molecules currently in use has not yet been fully established, and reliable long-term data on the incidence of CRC within the context of modern therapeutic strategies remain limited [55,69]. Several key gaps in our understanding therefore require further investigation. Prospective studies are needed to define optimal, risk-adjusted surveillance intervals, particularly in low-risk patients and the elderly, and to establish standardised criteria for discontinuing surveillance. Further research should validate molecular biomarkers and risk prediction models capable of complementing current clinical and endoscopic risk stratification. Prospective comparative studies are also needed to clarify the relative effectiveness of current endoscopic imaging strategies for the detection of dysplasia. Finally, long-term studies should determine whether prolonged control of inflammation using modern, advanced therapies results in a measurable reduction in the incidence of CRC.

## Figures and Tables

**Table 1:** Comparative performance of endoscopic imaging modalities for dysplasia surveillance in IBD.

Endoscopic Modality	Reported Sensitivity	Reported Specificity	Comparative Evidence	Main Advantages	Main Limitations
**HD** **-WLE**	94–100% [30]	85% [30]	Reference comparator; HD-DCE vs. HD-WLE: RR 1.42 (95% CI 1.02–1.98) [28]	Widely available; no dye application; high-resolution imaging	Subtle or flat lesions may still be difficult to detect; operator dependent
**DCE**	63–100% [30]	90–98% [30]	May detect more dysplasia than HD-WLE: RR 1.42 (95% CI 1.02–1.98) [28]	Enhanced mucosal contrast; improved lesion delineation	Dye application; greater procedural complexity and time
**VCE**	NBI: 50–100%; i-SCAN: 72% [30]	i-SCAN: 92%; not consistently reported for NBI [30].	Comparable performance in randomized evidence [26]; HD-VCE vs. HD-WLE: RR 0.99 (95% CI 0.69–1.43) [28]	No dye application; rapid activation; easy integration into routine colonoscopy	Performance varies across platforms; heterogeneous diagnostic-accuracy evidence

**DCE:** dye-based chromoendoscopy; **HD-DCE:** high-definition dye-based chromoendoscopy; **HD-VCE:** high-definition virtual chromoendoscopy; **HD-WLE:** high-definition white-light endoscopy; **IBD:** inflammatory bowel disease; **NBI:** narrow-band imaging; **RR:** risk ratio; **VCE:** virtual chromoendoscopy.

**Table 2:** Practical Framework for CRC Prevention in Patients with Colonic IBD.

Step	Clinical Assessment	Key Decision Point	Recommended Action
1	Confirm colonic involvement	Extent (proctitis vs. left-sided vs. extensive)	Identify eligibility for surveillance program [20,22]
2	Define disease duration	≥8 years colitis or PSC at diagnosis	Start surveillance at 8 years (or immediately in PSC) [20,22]
3	Risk stratification	PSC, family history for CRC, strictures, previous dysplasia, persistent endoscopic/histologic inflammatory activity, disease extension	Categorize into low/intermediate/high risk [15,20]
4	Surveillance interval	Low vs. intermediate vs. high CRC risk	Guideline-specific, risk-adapted surveillance: generally 1 year for high-risk patients, 2–3 years for intermediate-risk patients, and longer intervals for low-risk patients, with variation across ECCO, AGA, and BSG recommendations [20,22,27,63]
5	Endoscopic technique	Availability/expertise	High-definition colonoscopy with chromoendoscopy (dye-based or virtual) and targeted biopsies; consider additional random biopsies in high-risk settings (PSC, prior dysplasia, etc.) [20,22,27,63]
6	Dysplasia detected?	Visible vs. invisible; LGD vs. HGD	Visible dysplasia: endoscopic resection if well delineated and resectable; invisible dysplasia: repeat expert high-definition chromoendoscopy; invisible multifocal LGD/invisible HGD/unresectable visible dysplasia: consider colectomy [19,20]
7	Inflammation monitoring and disease control	Evidence of persistent or recurrent inflammation	Monitor activity using PROs, biomarkers (FC, CRP), IUS (or cross-sectional imaging), endoscopy when indicated, and optimize therapy to maintain objective remission and reduce cumulative inflammatory burden [27,63]
8	Reassessment of surveillance continuation	Age, comorbidity, prior neoplasia, recent colonoscopy	Reassess the need for continued surveillance individually, considering life expectancy and overall risk–benefit balance.

**AGA:** American Gastroenterological Association; **BSG:** British Society of Gastroenterology; **CRC:** Colorectal Cancer; **CRP:** C-Reactive Protein; **ECCO:** European Crohn’s and Colitis Organisation; **FC:** Fecal Calprotectin; **HGD:** High-Grade Dysplasia; **IBD:** Inflammatory Bowel Disease; **IUS:** Intestinal Ultrasound; **LGD:** Low-Grade Dysplasia; **PROs:** Patient-Reported Outcomes; **PSC:** Primary Sclerosing Cholangitis.

## Data Availability

No new data were created or analyzed in this study. Data sharing is not applicable to this article.

## References

[1] Marabotto E., Kayali S., Buccilli S., Levo F., Bodini G., Giannini E.G., Savarino V., Savarino E.V. (2022). Colorectal Cancer in Inflammatory Bowel Diseases: Epidemiology and Prevention: A Review. Cancers.

[2] Sato Y., Tsujinaka S., Miura T., Kitamura Y., Suzuki H., Shibata C. (2023). Inflammatory Bowel Disease and Colorectal Cancer: Epidemiology, Etiology, Surveillance, and Management. Cancers.

[3] Fanizza J., Bencardino S., Allocca M., Furfaro F., Zilli A., Parigi T.L., Fiorino G., Peyrin-Biroulet L., Danese S., D’Amico F. (2024). Inflammatory Bowel Disease and Colorectal Cancer. Cancers.

[4] Shah S.C., Itzkowitz S.H. (2022). Colorectal Cancer in Inflammatory Bowel Disease: Mechanisms and Management. Gastroenterology.

[5] Parigi T.L., Allocca M., Furfaro F., D’Amico F., Zilli A., Dal Buono A., Gabbiadini R., Bonovas S., Armuzzi A., Danese S. (2023). Treat-to-Target and Regular Surveillance of Inflammatory Bowel Disease Are Associated with Low Incidence and Early-Stage Detection of Malignancies: A Retrospective Cohort Study. Cancers.

[6] Eaden J.A., Abrams K.R., Mayberry J.F. (2001). The Risk of Colorectal Cancer in Ulcerative Colitis: A Meta-Analysis. Gut.

[7] Porter R.J., Arends M.J., Churchhouse A.M.D., Din S. (2021). Inflammatory Bowel Disease-Associated Colorectal Cancer: Translational Risks from Mechanisms to Medicines. J. Crohns Colitis.

[8] Yin Y., Wan J., Yu J., Wu K. (2023). Molecular Pathogenesis of Colitis-Associated Colorectal Cancer: Immunity, Genetics, and Intestinal Microecology. Inflamm. Bowel Dis..

[9] Hisamatsu T., Miyoshi J., Oguri N., Morikubo H., Saito D., Hayashi A., Omori T., Matsuura M. (2025). Inflammation-Associated Carcinogenesis in Inflammatory Bowel Disease: Clinical Features and Molecular Mechanisms. Cells.

[10] Chatila W.K., Walch H., Hechtman J.F., Moyer S.M., Sgambati V., Faleck D.M., Srivastava A., Tang L., Benhamida J., Ismailgeci D. (2023). Integrated Clinical and Genomic Analysis Identifies Driver Events and Molecular Evolution of Colitis-Associated Cancers. Nat. Commun..

[11] Hirsch D., Hardt J., Sauer C., Heselmeyer-Hadded K., Witt S.H., Kienle P., Ried T., Gaiser T. (2021). Molecular Characterization of Ulcerative Colitis-Associated Colorectal Carcinomas. Mod. Pathol..

[12] Du L., Kim J.J., Shen J., Chen B., Dai N. (2017). KRAS and TP53 Mutations in Inflammatory Bowel Disease-Associated Colorectal Cancer: A Meta-Analysis. Oncotarget.

[13] Lightner A.L., Vogler S., McMichael J., Jia X., Regueiro M., Qazi T., Steele S.R. (2021). Dysplastic Progression to Adenocarcinoma Is Equivalent in Ulcerative Colitis and Crohn’s Disease. J. Crohns Colitis.

[14] Clarke W.T., Feuerstein J.D. (2019). Colorectal Cancer Surveillance in Inflammatory Bowel Disease: Practice Guidelines and Recent Developments. World J. Gastroenterol..

[15] Lakatos P.-L., Lakatos L. (2008). Risk for Colorectal Cancer in Ulcerative Colitis: Changes, Causes and Management Strategies. World J. Gastroenterol..

[16] Samadder N.J., Valentine J.F., Guthery S., Singh H., Bernstein C.N., Leighton J.A., Wan Y., Wong J., Boucher K., Pappas L. (2019). Family History Associates With Increased Risk of Colorectal Cancer in Patients With Inflammatory Bowel Diseases. Clin. Gastroenterol. Hepatol..

[17] Wijnands A.M., de Jong M.E., Lutgens M.W.M.D., Hoentjen F., Elias S.G., Oldenburg B. (2021). Dutch Initiative on Crohn and Colitis (ICC) Prognostic Factors for Advanced Colorectal Neoplasia in Inflammatory Bowel Disease: Systematic Review and Meta-Analysis. Gastroenterology.

[18] Mahmoud R., Shah S.C., Ten Hove J.R., Torres J., Mooiweer E., Castaneda D., Glass J., Elman J., Kumar A., Axelrad J. (2019). No Association Between Pseudopolyps and Colorectal Neoplasia in Patients With Inflammatory Bowel Diseases. Gastroenterology.

[19] Laine L., Kaltenbach T., Barkun A., McQuaid K.R., Subramanian V., Soetikno R. (2015). SCENIC Guideline Development Panel SCENIC International Consensus Statement on Surveillance and Management of Dysplasia in Inflammatory Bowel Disease. Gastroenterology.

[20] Murthy S.K., Feuerstein J.D., Nguyen G.C., Velayos F.S. (2021). AGA Clinical Practice Update on Endoscopic Surveillance and Management of Colorectal Dysplasia in Inflammatory Bowel Diseases: Expert Review. Gastroenterology.

[21] Gordon H., Biancone L., Fiorino G., Katsanos K.H., Kopylov U., Al Sulais E., Axelrad J.E., Balendran K., Burisch J., de Ridder L. (2023). ECCO Guidelines on Inflammatory Bowel Disease and Malignancies. J. Crohns Colitis.

[22] East J.E., Gordon M., Nigam G.B., Sinopoulou V., Bateman A.C., Din S., Iacucci M., Kabir M., Lamb C.A., Wilson A. (2026). British Society of Gastroenterology Guidelines on Colorectal Surveillance in Inflammatory Bowel Disease. Gut.

[23] Axelrad J.E., Cross R.K. (2023). Surveillance for Colorectal Neoplasia in Inflammatory Bowel Disease: When to Stop. Am. J. Gastroenterol..

[24] Subramanian V., Ramappa V., Telakis E., Mannath J., Jawhari A.U., Hawkey C.J., Ragunath K. (2013). Comparison of High Definition with Standard White Light Endoscopy for Detection of Dysplastic Lesions during Surveillance Colonoscopy in Patients with Colonic Inflammatory Bowel Disease. Inflamm. Bowel Dis..

[25] Carballal S., Maisterra S., López-Serrano A., Gimeno-García A.Z., Vera M.I., Marín-Garbriel J.C., Díaz-Tasende J., Márquez L., Álvarez M.A., Hernández L. (2018). Real-Life Chromoendoscopy for Neoplasia Detection and Characterisation in Long-Standing IBD. Gut.

[26] Iacucci M., Kaplan G.G., Panaccione R., Akinola O., Lethebe B.C., Lowerison M., Leung Y., Novak K.L., Seow C.H., Urbanski S. (2018). A Randomized Trial Comparing High Definition Colonoscopy Alone With High Definition Dye Spraying and Electronic Virtual Chromoendoscopy for Detection of Colonic Neoplastic Lesions During IBD Surveillance Colonoscopy. Am. J. Gastroenterol..

[27] Kucharzik T., Taylor S., Allocca M., Burisch J., Ellul P., Iacucci M., Maaser C., Baldin P., Bhatnagar G., Ben-Horin S. (2025). ECCO-ESGAR-ESP-IBUS Guideline on Diagnostics and Monitoring of Patients with Inflammatory Bowel Disease: Part 1. J. Crohns Colitis.

[28] Sinopoulou V., Nigam G.B., Gordon M., Ganeshan M., Tokonyai M.R., Dolwani S., Iacucci M., Rutter M., Subramanian V., Wilson A. (2025). Comparative Efficacy and Safety of Endoscopic Modalities for Colorectal Cancer Screening in Inflammatory Bowel Disease: A Systematic Review and Network Meta-Analysis. Clin. Gastroenterol. Hepatol..

[29] Te Groen M., Derks M., den Broeder N., Peters C., Dijkstra G., de Vries A., Romkens T., Horjus C., de Boer N., de Jong M. (2024). Quality of Surveillance Impacts the Colitis-Associated Advanced Neoplasia Risk: A Multicenter Case-Control Study. Clin. Gastroenterol. Hepatol..

[30] Iannone A., Ruospo M., Wong G., Principi M., Barone M., Strippoli G.F.M., Di Leo A. (2017). Chromoendoscopy for Surveillance in Ulcerative Colitis and Crohn’s Disease: A Systematic Review of Randomized Trials. Clin. Gastroenterol. Hepatol..

[31] Watanabe T., Ajioka Y., Mitsuyama K., Watanabe K., Hanai H., Nakase H., Kunisaki R., Matsuda K., Iwakiri R., Hida N. (2016). Comparison of Targeted vs Random Biopsies for Surveillance of Ulcerative Colitis-Associated Colorectal Cancer. Gastroenterology.

[32] Bisschops R., East J.E., Hassan C., Hazewinkel Y., Kamiński M.F., Neumann H., Pellisé M., Antonelli G., Bustamante Balen M., Coron E. (2019). Advanced Imaging for Detection and Differentiation of Colorectal Neoplasia: European Society of Gastrointestinal Endoscopy (ESGE) Guideline—Update 2019. Endoscopy.

[33] Rutter M., Saunders B., Wilkinson K., Rumbles S., Schofield G., Kamm M., Williams C., Price A., Talbot I., Forbes A. (2004). Severity of Inflammation Is a Risk Factor for Colorectal Neoplasia in Ulcerative Colitis. Gastroenterology.

[34] Choi P.M., Nugent F.W., Schoetz D.J., Silverman M.L., Haggitt R.C. (1993). Colonoscopic Surveillance Reduces Mortality from Colorectal Cancer in Ulcerative Colitis. Gastroenterology.

[35] van Schaik F.D.M., Offerhaus G.J.A., Schipper M.E.I., Siersema P.D., Vleggaar F.P., Oldenburg B. (2009). Endoscopic and Pathological Aspects of Colitis-Associated Dysplasia. Nat. Rev. Gastroenterol. Hepatol..

[36] Pimentel-Nunes P., Libânio D., Bastiaansen B.A.J., Bhandari P., Bisschops R., Bourke M.J., Esposito G., Lemmers A., Maselli R., Messmann H. (2022). Endoscopic Submucosal Dissection for Superficial Gastrointestinal Lesions: European Society of Gastrointestinal Endoscopy (ESGE) Guideline—Update 2022. Endoscopy.

[37] Matsumoto K., Oka S., Tanaka S., Tanaka H., Boda K., Yamashita K., Sumimoto K., Ninomiya Y., Arihiro K., Shimamoto F. (2021). Long-Term Outcomes after Endoscopic Submucosal Dissection for Ulcerative Colitis-Associated Dysplasia. Digestion.

[38] Manta R., Zullo A., Telesca D.A., Castellani D., Germani U., Reggiani Bonetti L., Conigliaro R., Galloro G. (2021). Endoscopic Submucosal Dissection for Visible Dysplasia Treatment in Ulcerative Colitis Patients: Cases Series and Systematic Review of Literature. J. Crohns Colitis.

[39] Bak M.T.J., Albéniz E., East J.E., Coelho-Prabhu N., Suzuki N., Saito Y., Matsumoto T., Banerjee R., Kaminski M.F., Kiesslich R. (2023). Endoscopic Management of Patients with High-Risk Colorectal Colitis-Associated Neoplasia: A Delphi Study. Gastrointest. Endosc..

[40] Rubens M., Smith R. (2024). Management of Dysplasia in Inflammatory Bowel Disease. Clin. Colon Rectal Surg..

[41] Fumery M., Dulai P.S., Gupta S., Prokop L.J., Ramamoorthy S., Sandborn W.J., Singh S. (2017). Incidence, Risk Factors, and Outcomes of Colorectal Cancer in Patients With Ulcerative Colitis With Low-Grade Dysplasia: A Systematic Review and Meta-Analysis. Clin. Gastroenterol. Hepatol..

[42] De Jong M.E., Van Tilburg S.B., Nissen L.H.C., Kievit W., Nagtegaal I.D., Horjus C.S., Römkens T.E.H., Drenth J.P.H., Hoentjen F., Derikx L.A.A.P. (2019). Long-Term Risk of Advanced Neoplasia After Colonic Low-Grade Dysplasia in Patients with Inflammatory Bowel Disease: A Nationwide Cohort Study. J. Crohns Colitis.

[43] Holubar S.D., Lightner A.L., Poylin V., Vogel J.D., Gaertner W., Davis B., Davis K.G., Mahadevan U., Shah S.A., Kane S.V. (2021). The American Society of Colon and Rectal Surgeons Clinical Practice Guidelines for the Surgical Management of Ulcerative Colitis. Dis. Colon Rectum.

[44] Choi C.-H.R., Al Bakir I., Ding N.-S.J., Lee G.-H., Askari A., Warusavitarne J., Moorghen M., Humphries A., Ignjatovic-Wilson A., Thomas-Gibson S. (2019). Cumulative Burden of Inflammation Predicts Colorectal Neoplasia Risk in Ulcerative Colitis: A Large Single-Centre Study. Gut.

[45] Turner D., Ricciuto A., Lewis A., D’Amico F., Dhaliwal J., Griffiths A.M., Bettenworth D., Sandborn W.J., Sands B.E., Reinisch W. (2021). STRIDE-II: An Update on the Selecting Therapeutic Targets in Inflammatory Bowel Disease (STRIDE) Initiative of the International Organization for the Study of IBD (IOIBD): Determining Therapeutic Goals for Treat-to-Target Strategies in IBD. Gastroenterology.

[46] Gupta R.B., Harpaz N., Itzkowitz S., Hossain S., Matula S., Kornbluth A., Bodian C., Ullman T. (2007). Histologic Inflammation Is a Risk Factor for Progression to Colorectal Neoplasia in Ulcerative Colitis: A Cohort Study. Gastroenterology.

[47] Fousekis F.S., Mpakogiannis K., Filis P., Skamnelos A., Christodoulou D.K., Mauri D., Katsanos K.H. (2025). Exploring Chemoprevention in Colorectal Cancer for Patients with Inflammatory Bowel Disease: Mechanisms of Action and Clinical Aspects. Cancers.

[48] Rachma B., Pamungkas R.P., Sandra D.Y., Sutanto H., Fetarayani D. (2025). The Immunological Nexus between Inflammatory Bowel Disease and Colorectal Cancer: A Molecular Perspective. Clin. Immunol. Commun..

[49] Chapman C.G., Rubin D.T. (2014). The Potential for Medical Therapy to Reduce the Risk of Colorectal Cancer and Optimize Surveillance in Inflammatory Bowel Disease. Gastrointest. Endosc. Clin. N. Am..

[50] Bonovas S., Fiorino G., Lytras T., Nikolopoulos G., Peyrin-Biroulet L., Danese S. (2017). Systematic Review with Meta-Analysis: Use of 5-Aminosalicylates and Risk of Colorectal Neoplasia in Patients with Inflammatory Bowel Disease. Aliment. Pharmacol. Ther..

[51] Gisbert J.P., Chaparro M., Verstockt B., Kienle P., Minozzi S., Allocca M., Atreya R., Avellaneda N., Barreiro-de Acosta M., Battat R. (2026). ECCO Guidelines on Therapeutics in Ulcerative Colitis: Medical Treatment. J. Crohns Colitis.

[52] Jess T., Lopez A., Andersson M., Beaugerie L., Peyrin-Biroulet L. (2014). Thiopurines and Risk of Colorectal Neoplasia in Patients with Inflammatory Bowel Disease: A Meta-Analysis. Clin. Gastroenterol. Hepatol..

[53] Gordillo J., Cabré E., Garcia-Planella E., Ricart E., Ber-Nieto Y., Márquez L., Rodríguez-Moranta F., Ponferrada Á., Vera I., Gisbert J.P. (2015). Thiopurine Therapy Reduces the Incidence of Colorectal Neoplasia in Patients with Ulcerative Colitis. Data from the ENEIDA Registry. J. Crohns Colitis.

[54] Peyrin-Biroulet L., Khosrotehrani K., Carrat F., Bouvier A.-M., Chevaux J.-B., Simon T., Carbonnel F., Colombel J.-F., Dupas J.-L., Godeberge P. (2011). Increased Risk for Nonmelanoma Skin Cancers in Patients Who Receive Thiopurines for Inflammatory Bowel Disease. Gastroenterology.

[55] Alkhayyat M., Abureesh M., Gill A., Khoudari G., Abou Saleh M., Mansoor E., Regueiro M. (2021). Lower Rates of Colorectal Cancer in Patients With Inflammatory Bowel Disease Using Anti-TNF Therapy. Inflamm. Bowel Dis..

[56] Charkaoui M., Hajage D., Tubach F., Beaugerie L., Kirchgesner J. (2022). Impact of Anti-Tumour Necrosis Factor Agents on the Risk of Colorectal Cancer in Patients with Ulcerative Colitis: Nationwide French Cohort Study. J. Crohns Colitis.

[57] Peery A.F., Kelly C.R., Kao D., Vaughn B.P., Lebwohl B., Singh S., Imdad A., Altayar O., AGA Clinical Guidelines Committee (2024). Electronic address: Clinicalpractice@gastro.org AGA Clinical Practice Guideline on Fecal Microbiota-Based Therapies for Select Gastrointestinal Diseases. Gastroenterology.

[58] Barberio B., Massimi D., Cazzagon N., Zingone F., Ford A.C., Savarino E.V. (2021). Prevalence of Primary Sclerosing Cholangitis in Patients With Inflammatory Bowel Disease: A Systematic Review and Meta-Analysis. Gastroenterology.

[59] Pitoni D., Dal Buono A., Gabbiadini R., Ronca V., Colapietro F., Pugliese N., Ribaldone D.G., Bezzio C., Lleo A., Armuzzi A. (2025). Navigating Neoplasm Risk in Inflammatory Bowel Disease and Primary Sclerosing Cholangitis. Cancers.

[60] Abdalla M., Eberhardson M., Landerholm K., Andersson R.E., Myrelid P. (2026). Impact of Inflammatory Bowel Disease and Primary Sclerosing Cholangitis on Colorectal Cancer Risk: National Cohort Study. Clin. Gastroenterol. Hepatol..

[61] Schiavone S.C., Biancone L., Fiorillo M., Divizia A., Mancone R., Neri B. (2025). Colitis-Associated Dysplasia in Inflammatory Bowel Disease: Features and Endoscopic Management. Cancers.

[62] Khan I., Belkovsky M., Gorgun E. (2024). The Old: Is There Any Role for Screening Colonoscopy after the Age of 75? The Surgeon’s Perspective. Clin. Colon Rectal Surg..

[63] Yanai H., Feakins R., Allocca M., Burisch J., Ellul P., Iacucci M., Maaser C., Zilli A., Zidar N., Wilkens R. (2025). ECCO-ESGAR-ESP-IBUS Guideline on Diagnostics and Monitoring of Patients with Inflammatory Bowel Disease: Part 2. J. Crohns Colitis.

[64] Lutgens M.W.M.D., van Oijen M.G.H., van der Heijden G.J.M.G., Vleggaar F.P., Siersema P.D., Oldenburg B. (2013). Declining Risk of Colorectal Cancer in Inflammatory Bowel Disease: An Updated Meta-Analysis of Population-Based Cohort Studies. Inflamm. Bowel Dis..

[65] Dell’Anna G., Mandarino F., Centanni L., Lodola I., Fanizza J., Fasulo E., Bencardino S., Fuccio L., Facciorusso A., Donatelli G. (2025). Transforming Gastrointestinal Diagnosis with Molecular Endoscopy: Challenges and Opportunities. Int. J. Mol. Sci..

[66] Iacucci M., Santacroce G., Yasuharu M., Ghosh S. (2025). Artificial Intelligence-Driven Personalized Medicine: Transforming Clinical Practice in Inflammatory Bowel Disease. Gastroenterology.

[67] Bencardino S., Lodola I., Centanni L., Mandarino F.V., Fanizza J., Furfaro F., D’Amico F., Fuccio L., Bruni A., Facciorusso A. (2025). Artificial Intelligence in Advanced Endoscopic Imaging: Transforming Optical Diagnosis in Gastroenterology. Front. Med..

[68] Takenaka K., Kawamoto A., Okamoto R., Watanabe M., Ohtsuka K. (2022). Artificial Intelligence for Endoscopy in Inflammatory Bowel Disease. Intest. Res..

[69] Card T., Ungaro R., Bhayat F., Blake A., Hantsbarger G., Travis S. (2020). Vedolizumab Use Is Not Associated with Increased Malignancy Incidence: GEMINI LTS Study Results and Post-Marketing Data. Aliment. Pharmacol. Ther..

